# Gene Therapy: A Possible Alternative to CFTR Modulators?

**DOI:** 10.3389/fphar.2021.648203

**Published:** 2021-04-21

**Authors:** J. Mercier, M. Ruffin, H. Corvol, L. Guillot

**Affiliations:** ^1^ Sorbonne Université, Inserm, Centre de Recherche, Saint Antoine, F-75012, Paris, France; ^2^ Pneumologie Pédiatrique, APHP, Hôpital Trousseau, Paris, France

**Keywords:** cystic fibrosis, gene therapy, ivacaftor, lumacaftor, tezacaftor, personalized medicine

## Abstract

Cystic fibrosis (CF) is a rare genetic disease that affects several organs, but lung disease is the major cause of morbidity and mortality. The gene responsible for CF, the *CFTR* (Cystic Fibrosis Transmembrane Conductance Regulator) gene, has been discovered in 1989. Since then, gene therapy i.e., defective gene replacement by a functional one, remained the ultimate goal but unfortunately, it has not yet been achieved. However, patients care and symptomatic treatments considerably increased CF patients’ life expectancy ranging from 5 years old in the 1960s to 40 today. In the last decade, research works on CFTR protein structure and activity led to the development of new drugs which, by readdressing CFTR to the plasma membrane (correctors) or by enhancing its transport activity (potentiators), allow, alone or in combination, an improvement of CF patients’ lung function and quality of life. While expected, it is not yet known whether taking these drugs from an early age and for years will improve the quality of life of CF patients in the long term and further increase their life expectancy. Besides, these molecules are not available (specific variants of *CFTR*) or accessible (national health policies) for all patients and there is still no curative treatment. Another alternative that could benefit from new technologies, such as gene therapy, is therefore still attractive, although it is not yet offered to patients. Faced with the development of new CFTR correctors and potentiators, the question arises as to whether there is still a place for gene therapy and this is discussed in this perspective.

## Introduction

Cystic fibrosis (CF) is a rare genetic disease caused by pathogenic variants in the *CFTR* (Cystic Fibrosis Transmembrane Conductance Regulator) gene, which encodes for a chloride channel expressed ubiquitously within epithelia. As a result, ionic and hydric imbalances across epithelia are observed in several organs, affecting their function. Specifically, manifestations can occur in the pancreas, liver, kidneys, and intestine, but lung disease is the main cause of morbidity and mortality of CF patients.

Since the *CFTR* gene discovery in 1989, more than 2000 variants of the *CFTR* gene have been identified. They were classified into six classes depending on their consequences on the CFTR protein. CF patients’ life expectancy increased considerably between the 1960s and the beginning of the 21^st^ century mainly due to symptomatic treatment development and to patient management standardization. However, even if new promising drugs (called “CFTR modulators”) directly targeting the CFTR protein are already available for some CF patients, there is still no curative treatment. Gene therapy, as defined by the delivery of a wild-type *CFTR* gene in cells, might be this curative treatment but is not yet available.

This perspective briefly describes the latest development of CFTR modulators and *CFTR* gene delivery strategies and discusses whether gene therapy is a still relevant alternative to be considered with respect to these promising molecules.

### CFTR Modulators

In the last decade, new drugs called “CFTR modulators” addressing the direct consequences of *CFTR* variants on the CFTR protein have been identified thanks to high-throughput screening (extensively reviewed in (Lopes-Pacheco, 2019)). The two major types of CFTR modulators are correctors and potentiators. Correctors are intended to improve CFTR trafficking to the plasma membrane, while potentiators are designed to increase CFTR channel conductance. Thus, correctors and potentiators can only be effective on certain classes of *CFTR* variants i.e., those affecting CFTR protein trafficking (class II) and conductance (class III and IV). *CFTR* class I variants, that result in the absence of protein synthesis, and *CFTR* class V and VI variants, that lead to decreased CFTR protein quantity production, are excluded from modulator therapies. CFTR modulators approval represents a breakthrough in CF care because a significant improvement in patient’s lung function (evaluated by measurements of FEV1 (forced expiratory volume in 1 s)) has been observed (Table 1).

**TABLE 1:** CFTR modulators available for the treatment of CF patients. ppFEV1: percent-predicted Forced Expiratory Volume in 1 s; m.o.: months old; y.o.: years old. *only initial Phase III studies are cited. [detailed in (Lopes-Pacheco, 2019)].

Molecules	First approval date	Eligibility age	Indication*	Absolute change in ppFEV1
Ivacaftor	2012	≥6 m.o.	G551D/other Ramsey et al. (2011)	10.6%
			Class III variants/Other Davies et al. (2013)	12.5%
Lumacaftor/ivacaftor	2015	≥2 y.o.	F508del/F508del Wainwright et al. (2015)	2.8%
Tezacaftor/ivacaftor	2018	≥6 y.o.	F508del/F508del Taylor-Cousar et al. (2017)	4%
			F508del/residual function variant in *trans* Rowe et al. (2017)	6.8%
Elexacaftor/tezacaftor/ivacaftor	2019	≥12 y.o.	F508del/minimal function variant in *trans* Middleton et al. (2019)	13.8%
			F508del/F508del Heijerman et al. (2019)	10.4%

Ivacaftor, the first potentiator designed to target the G551D gating variant (the most prevalent *CFTR* class III gating variant), showed in phase 3 clinical trial that patients gain more than 10% of FEV1 (Ramsey et al., 2011). Ivacaftor was then validated for other *CFTR* class III gating variants. A few years after, modulators targeting the most common *CFTR* variant called F508del (a class II variant) emerged. The combination of the corrector lumacaftor with the potentiator ivacaftor was approved for patient homozygotes for the F508del variant but showed limited FEV1 improvement. New correctors and combinations emerged further, which have more interesting effects on FEV1, allowing to address patients carrying only one F508del variant (Table 1). The latest combination (elexacaftor/tezacaftor/ivacaftor) showed indeed impressive results with a 14% increase in FEV1 (Heijerman et al., 2019; Middleton et al., 2019). Even if the level of increased lung function remains the principal measurement of treatment efficiency, these modulators have also been shown to be beneficial for other clinical parameters including increased body-mass index, improved life quality, and decreased exacerbations frequency.

Altogether, based on their age and genotype, around 80% of the patients reported in the Cystic Fibrosis Foundation Patient Registry are eligible for CFTR modulators (https://www.cff.org/Research/Researcher-Resources/Patient-Registry/). According to this registry, 10% of the patients are still too young to be eligible for CFTR modulators; and the remaining 10% carry *CFTR* variants not currently known to be responsive to modulators or have unknown or incomplete genotypes. Among these patients, it has been estimated that 7% may require genetic-based therapy.

In this new promising era, some other concerns remain. For example, it should be noted that the hindsight on these treatments is limited, which makes it difficult to assess their long-term efficacy and tolerance. According to national health policies, the cost of these treatments might also be an obstacle to their access for all CF patients. Finally, as shown in a recent retrospective study, among 845 CF patients who initiated lumacaftor/ivacaftor, 22.8% fully or temporarily discontinued their treatment mostly because of respiratory adverse events (Burgel et al., 2020). This could result in limiting the total number of patients who can benefit from these treatments.

The latest generation of modulator, the triple therapy, elexacaftor/tezacaftor/ivacaftor, showed impressive results leading us to question in this perspective whether gene therapy is still attractive.

## Gene Therapy: What’s Up?

Gene therapy consists of introducing a functional gene into host cells to replace a defective gene. Theoretically, this could be a perfect match for the needs of a monogenic disease as CF and could lead to a universal treatment for CF patients. Especially since Johnson and colleagues stated in 1992 that 6–10% of CFTR-corrected cells are sufficient to observe a therapeutic effect *in vitro* (Johnson et al., 1992), we can speculate that correction of all CF airway cells may not be mandatory to observe the same effect *in vivo*. However, the race for CF gene therapy turned out to be more challenging than expected so far.

### Lungs: Barriers to Delivery

The lung represents an organ of choice for the delivery of organ-specific treatments due to its ease of access. Therefore, aerosol administration has been widely used in previous clinical trials of pulmonary gene therapy as reviewed by Resnier et al. (Resnier et al., 2016). However, the major difficulty encountered in these trials is the delivery of the gene into airway epithelial cells. Indeed, mucus, mucociliary clearance, and lung immune responses complicate the entry of the gene transfer agent into target cells, and the few that succeed encounters a new obstacle: the nuclear membrane (Yonemitsu et al., 2000; Stern et al., 2003; Schuster et al., 2014; Xia et al., 2014). Moreover, since the airway epithelium renews itself gradually, another challenge is to target progenitor cells to obtain a permanent treatment and avoid repeated required deliveries (Gill and Hyde, 2014).

### CFTR Gene Delivery Methods: Advantages and Limitations

**FIGURE 1:**
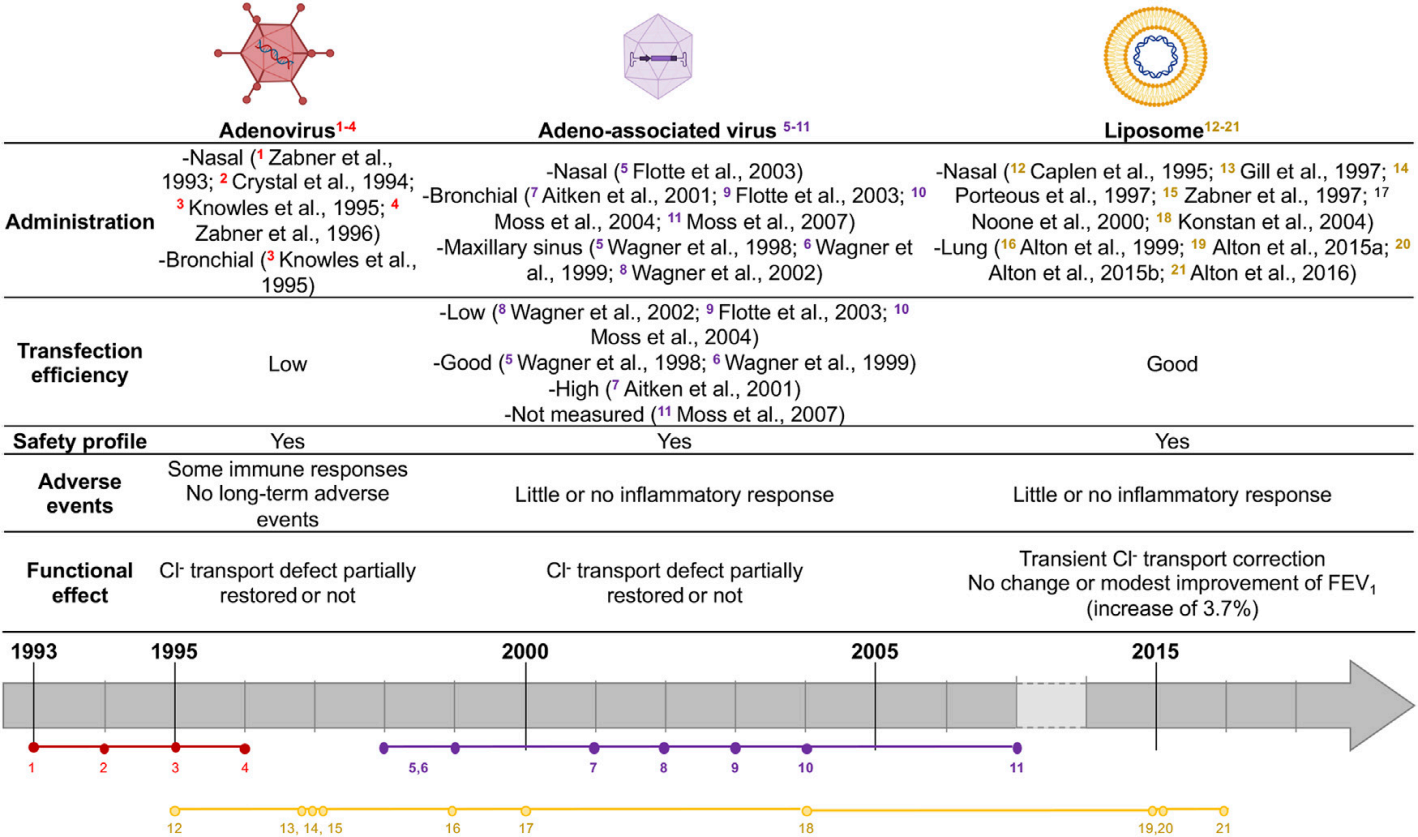
Summary of CF gene therapy clinical trials. Colors represent vector agent used (red: Ad, purple: AAV, yellow: liposome). Vectors agent made with biorender: https://biorender.com.

Only 1 year after the *CFTR* gene discovery in 1989 (Kerem et al., 1989; Riordan et al., 1989; Rommens et al., 1989), two groups independently showed that it was possible to produce a functional CFTR protein by using a viral vector to introduce the full-length *CFTR* gene *in vitro* (Drumm et al., 1990; Rich et al., 1990). This proof of concept paved the way for the beginning of CF gene therapy clinical trials (Figure 1). To efficiently introduce a normal *CFTR* gene into cells, various delivery strategies have been developed for three decades, including viral and non-viral vector systems, and are briefly presented after (recently reviewed in detail by (Cooney et al., 2018; Schneider-Futschik, 2019; Yan et al., 2019)).

#### Viral Vectors

Viruses have a high natural capacity to infect host cells and were modified by scientists to decrease their pathogenicity. Thus, recombinant vectors expressing a foreign antigenic protein or a functional protein were created and used for vaccines or gene therapy, respectively.

#### Recombinant Adenovirus Vector

Recombinant Adenovirus-based vectors (rAd-vector) were the first viral vectors used in CF gene therapy clinical trials. They are non-enveloped viruses with a linear and double-stranded DNA genome. Commonly, two serotypes are observed beyond Ad - type 2 (Ad2) or type 5 (Ad5) which allow host-cell binding. *In vivo* studies showed that Ad-vectors were transduced in all airway epithelial cell types (Mastrangeli et al., 1993) and also in submucosal glands (Pilewski et al., 1995). The first use of Ad-vectors with *CFTR* gene in CF gene therapy clinical trial showed that their delivery was safe, with little or no immune response and a partial correction of Cl^−^ transport defect (Zabner et al., 1993; Zabner et al., 1996). Crystal et al. showed that rAd-CFTR protein was expressed in the airway epithelium 4 days after intra-bronchial administration. They tested doses up to 2 × 10^9^ pfu, which led to short-term adverse events such as transient systemic inflammation (Crystal et al., 1994). No other adverse event was reported after a 6-months follow-up. Another trial showed no evidence of CFTR functional defect correction, probably due to inflammatory responses (Knowles et al., 1995). Thus, rAd-vectors are capable of transducing and occasionally correcting Cl^−^ transport defect in CF human airway epithelial cells. Yet, some inflammatory responses were observed. Besides, rAd-vector DNA does not integrate the host cell genome but rather persists within the cells as episomal DNA (Athanasopoulos et al., 2017). Finally, rAd-vector DNA is not replicated upon cell divisions, requiring multiple deliveries for patients.

#### Recombinant Adeno-Associated Virus Vector

Recombinant adeno-associated virus vectors (rAAV) are characterized by their ability to transduce both dividing and quiescent cells and by their high transduction efficiency in primary human airway (Yan et al., 2013). Those vectors are less immunogenic than Ad-vectors and were the most used vectors in CF gene therapy clinical trials, showing a strong safety profile record but failed in improving the lung function (Wagner et al., 1999; Wagner et al., 1998; Aitken et al., 2001; Wagner et al., 2002; Flotte et al., 2003; Moss et al., 2007) (Figure 1).

A limitation of rAAV vector is its packaging capacity limited to 4.8 kb (Blacklow et al., 1988; Wu et al., 2010). The small size of this vector may give it an advantage to enter cells more easily but considering that the size of the *CFTR* gene is 4.6 kb, there is almost no space left for regulatory sequences allowing *CFTR* gene expression enhancement. As reviewed by Cooney and coll., the shortening of the *CFTR* gene and other cassette sequences led to a generation of modified AAV vectors that expressed functional CFTR.

Moreover, rAAVs vectors genome integrates into the human genome (Kotin et al., 1991; Samulski et al., 1991; Kotin et al., 1992; Rivadeneira et al., 1998; Ding et al., 2003; Janovitz et al., 2014) and can also persist as episomal DNA in post-mitotic tissues when the replication protein rep is absent (Athanasopoulos et al., 2017). Besides, rAAV-mediated transduction has been proved to induce a long-lasting gene expression up to several months in pigs (Steines et al., 2016) and better rAAV vectors allowing an increased packaging capacity and higher tropism for airway epithelial cells are developed (Yan et al., 2019).

#### Helper-Dependent Adenovirus Vector

Helper-dependent adenovirus (Hd-Ad) vectors, which have had all viral genes deleted, were developed to circumvent inflammatory properties of rAd-vectors (Parks et al., 1996). With a 36 kb packaging capacity (Cots et al., 2013), they circumvent little packaging capacities of rAAV-vectors. Multiple serotypes of Hd-Ad vectors have been tested but serotype 5 showed effective transduction *in vivo* in airways basal cells in mouse and pig models and, *in vitro*, in primary human airway epithelial cell cultures (Cao et al., 2018; Palmer et al., 2020).

#### Lentivirus

Finally, the last type of viral vector used for CF gene therapy studies is lentiviral vector, which could allow a genomic integration and provide a long-term expression (Naldini et al., 1996) without repeated administrations. Even if LV vectors seem less likely to be destroyed by immune system than rAAVs, because of no-prexisting immunity (neutralizing antibodies), they activate the immune system as the other viral vectors (Nayak and Herzog, 2010). LV-mediated transgene integration site into the host genome can be multiple, extremely variable and potentially leading to proto-oncogenes activation. This property led to the interruption of a clinical trial in severe combined immunodeficiency patients (Hacein-Bey-Abina et al., 2003a; Hacein-Bey-Abina et al., 2003b; Fischer et al., 2004; Fischer and Cavazzana-Calvo, 2005; Hacein-Bey-Abina et al., 2008). In order to circumvent this, LV vectors design was then improved with self-inactivating (SIN) of long terminal repeats (LTRs), reducing the genotoxic risk of these strong enhancer-promoter sequences (Modlich and Baum, 2009; Milone and O'Doherty, 2018). Thus, lentiviruses still seem to be interesting vectors for CF gene therapy as some lentiviral vector pseudotypes demonstrated high vector production and apical tropism to airway epithelium *in vitro* and *in vivo* (Sinn et al., 2005; McKay et al., 2006). Plus, repeated administration was possible without blocking antibody immune responses (Sinn et al., 2008; Griesenbach et al., 2012) and LV efficiency was demonstrated in several animal models including mice, ferret and pigs (Oakland et al., 2013; Yan et al., 2015; Cooney et al., 2016). Recently, a study with a promising lentiviral vector developed by the United Kingdom Cystic Fibrosis Gene Therapy consortium showed a 14% airway cells transduction efficiency *in vitro*, low toxicity and an integration site profile supporting a future first-in-man trial (Alton et al., 2017).

Nevertheless, viral vectors use can be limited by several factors. Firstly, neutralizing antibodies to Ad or AAV, due to the immune response to previous natural infection, are frequently found in patients, rendering them ineligible for this kind of treatments (Erles et al., 1999). Secondly, viral vectors internalization into host cells requires receptors such as α_V_ integrins (Wickham et al., 1993), Coxsackie-Adenovirus Receptor (Bergelson et al., 1997), heparan sulfate glycosaminoglycans (Dechecchi et al., 2001) or fibroblast growth factor receptors (Duan et al., 1998) that are expressed on the basolateral membrane of bronchial epithelial cells, thus explaining the lack of transduction efficiency (Duan et al., 1998; Pickles et al., 1998; Marquez Loza et al., 2019). Pseudotyping viral vectors with proteins able to interact with apical membrane receptors of airway epithelial cells is thus required to enhance delivery efficiency.

#### Non-Viral Vectors

Non-viral vectors allow genomic material delivery into cells by direct administration as naked DNA or associated with different compounds. Those vectors have theoretically no size restrictions and have mainly three possible configurations: lipid-based, peptide-based or polymer-based delivery. They can carry plasmid or linear DNA as well as RNA molecules. A non-viral strategy for *CFTR* delivery was considered as an alternative to viral vectors. Indeed, several studies showed that cationic lipids were safe delivery vehicles for gene transfer (Kollen et al., 1996; Fasbender et al., 1998; McDonald et al., 1998), their main issue remaining the limited transgene delivery into the nucleus (Brisson et al., 1999). However, as recently reviewed (Cooney et al., 2018), several clinical trials using cationic lipids showed transient expression of vector related-*CFTR* for few days and partial restoration of nasal potential differences in CF patients without significant clinical effect on lung function (Caplen et al., 1995; Gill et al., 1997; Porteous et al., 1997; Zabner et al., 1997; Alton et al., 1999; Noone et al., 2000; Ruiz et al., 2001; Konstan et al., 2004). In 2015, Alton and colleagues reported an increase in FEV1 of 3.7% (0.07–7.25%) after using pGM169/GL67A cationic lipid (Alton et al., 2015b) in patients who received nebulized vector at 28 days intervals for 12 months (Alton et al., 2015a). However, as FEV1 improvements observed were only comparable to those obtained with some CFTR modulators, trial did not result in the current clinical application for CF patients. Alton et al. now focused on alternative lentiviral vectors candidates, such as the lentiviral vector based on simian immunodeficiency virus (SIV) pseudotyped with Sendaï virus envelope proteins Fusion (F) and Hemagglutinin-Neuraminidase (HN) (rSIV.F/HN), which retains 90–100% transduction efficiency and leads to a functional CFTR expression in preclinical models (Alton et al., 2016; Alton et al., 2017).

## Gene Therapy Still to Consider?

To date, CF gene therapy clinical trials have not been conclusive, with a weak effect observed on CF patients’ lung function, associated with important immune responses, which may render the treatment ineffective. Moreover, as mentioned previously, the recent developments of effective drugs such as the triple combination elexacaftor/tezacaftor/ivacaftor, which greatly improves patients’ lung function and quality of life, has given rise to the idea that “cystic fibrosis is almost cured”. Thus, it is therefore legitimate to wonder to what extent gene therapy still has a place in the treatment of CF. However, it should be remembered that the drugs currently on the market still leave aside at least 10% of CF patients without curative treatment, as highlighted by the Cystic Fibrosis Foundation patient registry. In addition, current CFTR modulators are not well tolerated by all eligible patients and real-life studies show that numerous patients stop their treatment. Besides, we do not have enough hindsight to predict effectiveness of these promising drugs in the long term.

Over the last 20 years, gene therapy has shown successful transient or permanent results for several diseases such as X-linked severe combined immune deficiency (Cavazzana-Calvo et al., 2000), adenosine deaminase deficiency (Cicalese et al., 2016), Leber congenital amaurosis (Cideciyan et al., 2013), hemophilia (Nathwani et al., 2011), severe beta-Thalassemia (Cavazzana-Calvo et al., 2010; Persons, 2010), but also Parkinson’s disease (Palfi et al., 2014) or leukemia, with 27 children who experienced complete remission after treatment (Maude et al., 2014). In 2012, the first viral gene-therapy treatment was approved in Europe for treatment of lipoprotein lipase deficiency (Yla-Herttuala, 2012) and showed good results even years later (Gaudet et al., 2016). Recently, gene therapy also showed promising results for Leber hereditary optic neuropathy by improving bilateral vision in patients over 96 weeks of follow-up after a unilateral intravitreal injection (Yu-Wai-Man et al., 2020). These successful trials were carried out on easily accessible hematopoietic stem cells or by direct vectors injections into specific tissues, which may explain previous trials failure in CF compared to other diseases.

In addition to wild-type *CFTR* gene delivery strategy, *CFTR* gene editing with CRISPR/Cas9 technology emerged as a promising therapeutic option for CF patients (Hodges and Conlon, 2019). New strategies are also currently tested in on-going trials, such as RESTORE-CF phase I/II clinical trial (http://ClinicalTrials.gov Identifier: NCT03375047), which evaluates safety and tolerability of MRT5005, an RNA-based therapy. RNA-based therapy has the advantage of not disturbing the genome, however would require repeated doses. Another promising strategy is to combine gene therapy with pluripotent stem cells (iPSCs). As a proof of concept, Hawkins et al. succeeded to derive airway basal cells from human iPSCs, to perform an engraftment and to allow epithelial regeneration *in vivo* using a tracheal xenograft model in immune-compromized mice, with similar structure and composition than *in vivo* airways (Hawkins et al., 2020). The long-term goal is to obtain a *de novo* generation of the full diversity of lung lineages and transplantable 3D lung tissues, that could be corrected by gene therapy and transferred into CF patients (Kotton, 2012).

In conclusion, CF gene therapy still has a bright future ahead. The main current challenge is to circumvent technical problems of transduction encountered in the lung. If this happens one day, not only will CF be truly cured, but the gene therapy might also be applied to other lung genetic diseases.
